# Relation between Apolipoprotein E Gene Polymorphism and Severity of Coronary Artery Disease in Acute Myocardial Infarction

**DOI:** 10.1155/2015/363458

**Published:** 2015-08-24

**Authors:** Zülküf Karahan, Murat Uğurlu, Berzal Uçaman, Ali Veysel Uluğ, İlyas Kaya, Kemal Çevik, Önder Öztürk, Hikmet Iyem

**Affiliations:** ^1^Cardiology, Gazi Yasargil Education and Research Hospital, 21070 Diyarbakır, Turkey; ^2^Cardiovascular Surgery, Gazi Yasargil Education and Research Hospital, 21070 Diyarbakır, Turkey

## Abstract

Apolipoprotein E (ApoE) is a plasma protein and associated with cholesterol transport system. In several studies, the relationship between ApoE gene polymorphism and severity of coronary artery disease (CAD) has been shown. However, the relationship between ApoE gene polymorphism and severity of CAD in patients with acute myocardial infarction (MI) has not been well known. The aim of this study is to investigate the relation between ApoE polymorphism and severity of CAD in patients with acute MI by using the Gensini Score. In this study, 138 patients were admitted to cardiology clinic with diagnosis of acute MI, and angiographic assessment was performed using the Gensini Score. Blood samples were obtained from all patients in the first day. The patients with ApoE34 genotype had high Gensini scores. Besides, the patients with E4 allele carriers were associated with high Gensini score compared with the patients without E4 allele carriers (*p*:0,22). The patients with E4 allele carriers were associated with higher LDL cholesterol and total cholesterol compared with the patients without E4 allele carriers (*p*:0,001 and *p*:0,03, resp.). There were no statistically significant differences between ApoE genotypes and severity of CAD by using the Gensini Score. But, the patients with E4 allele carriers were associated with high lipid levels.

## 1. Introduction

Myocardial infarction (MI) is the main cause of death in developed countries and is a multifactorial disease influenced by environmental factors and genetic mutations. Among other known reasons of MI, apolipoprotein E (ApoE) has been regarded as a potential genetic marker for coronary artery disease (CAD) [1–3].

ApoE is a serum glycoprotein of 34 kDa with 299 amino acids that serves as a ligand for cell-surface receptor uptake of chylomicrons and very low density lipoproteins (VLDL) on the liver and controls intestinal cholesterol absorption [4–6]. ApoE gene polymorphism includes three isoforms called ApoE2, ApoE3, and ApoE4 which give rise to six different genotypes [7]. The three isoforms are different in terms of properties such as affinity binding to ApoE, low density lipoprotein receptors, and lipoprotein particles [8]. Previous studies have shown that ApoE alleles have an effect on the lipid clearance and metabolism in humans. The ApoE2 allele has been shown to be associated with higher serum levels of ApoE, lower serum levels of LDL cholesterol (LDL-C), and lower risk of CAD [9]. The ApoE4 allele is associated with lower serum levels of ApoE, higher serum levels of total cholesterol (TC), LDL-C, and VLDL-C, and higher risk of CAD [10]. However, in a previous study by Ward et al., no association was found between ApoE genotypes and CAD [11]. Epidemiologic studies between ApoE gene polymorphism and cardiovascular outcomes have been shown to be inconsistent conclusions.

The aim of this study was to evaluate the relationship between ApoE gene polymorphism and severity of coronary artery disease in patients with acute MI by using the Gensini scores in the southeast region of Turkey. Its relationship with lipids and other cardiovascular risk factors is also examined and has not been studied in this region so far. The study protocol was approved by the local Ethics Committee and informed written consent was obtained from all patients.

## 2. Methods

### 2.1. Patients

This study enrolled 138 (107 males, 31 females) consecutive patients with AMI. All the patients underwent coronary angiography on admission. The diagnosis of AMI was based on the presence of at least two of the following: chest pain lasting longer than 30 minutes, rise in creatinine kinase myocardial band (CKMB), and new ST elevation of at least 0.1 mV in two or more contiguous precordial leads. Exclusion criteria were previous myocardial infarction, cardiogenic shock, chronic renal failure, unstable angina pectoris, and myocardial infarction with non-ST elevation. Predisposing factors for CAD like family history, hypertension, diabetes, and smoking were determined through interaction with the patients or from their clinical records.

Blood samples were taken from all patients on admission to the coronary care unit. Blood samples were drawn from antecubital vein and put into EDTA (5 mg/mL) tubes for lipid analysis. Total cholesterol, HDL-C, and triglyceride levels were measured by enzymatic colorimetric methods and LDL-C levels were calculated by using the Friedewald formula. Blood samples taken for genetic analysis were sent to the laboratory in complete blood count tubes. The genotypic structure was detected by CVD StripAssay method that works with a reverse hybridization principle followed by performing PCR.

All coronary angiograms were recorded using a Philips Allura Xper FD device (Philips Medical Systems Nederland B. V. Veldhoven, Netherlands). Standard coronary angiography with at least 2 views of the right coronary artery and 4 views of the left coronary artery was used. Two experienced cardiologists blinded to the clinical history and laboratory results of the patients performed the Gensini score, and discrepancies were solved by consensus. The intensity of coronary atherosclerosis was assessed by using the Gensini score [12] which grades the narrowing of the lumen of the coronary arteries as 1 for 1%–25% narrowing, 2 for 26%–50% narrowing, 4 for 51%–75% narrowing, 8 for 76%–90% narrowing, 16 for 91%–99% narrowing, and 32 for total occlusion. This score is then multiplied by a factor that takes into account the importance of the lesion's position in the coronary arterial tree, for example, 5 for the left main coronary artery, 2.5 for the proximal left anterior descending (LAD) coronary artery or proximal left circumflex (LCX) coronary artery, 1.5 for the midregion of LAD, and 1 for the distal LAD or middistal region of the LCX. The Gensini score was expressed as the total of the scores for all coronary arteries. In addition, the other angiographic variables were multivessel coronary artery and infarct-related artery (IRA).

### 2.2. Statistical Method

SPSS 18.0 (SPSS, Inc., Chicago, Illinois) was used for statistical analyses. The normality of the distribution of continuous variables was evaluated by the Kolmogorov-Smirnov test. For continuous variables, Kruskal Wallis test and Mann-Whitney test were used to test differences between groups. Two-tailed *p* values <0.05 were considered statistically significant. Continuous variables were expressed as mean ± SD, and categorical variables were expressed as percentages.

## 3. Results

In this study, mean age of the patients was 58 ± 10 years. Twenty-five percent of the patients had hypertension, 12% of the patients had diabetes mellitus (DM), 10% of the patients had a family history, and 52% of the patients were smokers. In the ApoE gene polymorphism isoforms, it was found that one (0,9%) of the patients had E22 genotype, 17 (12,3%) of the patients had E23 genotypes, 80 (57,9%) of the patients had E33 genotypes, and 40 (28,9%) of the patients had E34 genotypes. No significant relation was found between ApoE gene polymorphism and hypertension, diabetes mellitus, family history, and smoking in Table 1. There was no significant difference among ApoE gene isoforms for the Gensini score (*p*:0,13). But the patients with ApoE34 genotype had high Gensini scores. Besides, when compared with the patients without E4 allele carriers, the patients with E4 allele carriers had high Gensini score (*p*:0,22). The association between ApoE gene polymorphism and lipid profile was shown in Table 2. When compared with the patients without E4 allele carriers, the patients with E4 allele carriers had higher LDL cholesterol and total cholesterol (*p*:0,001 and *p*:0,03, resp.).

## 4. Discussion

Myocardial infarction is a multifactorial disease affected by environmental factors and genetic mutations. The present study investigated the effect of ApoE gene polymorphism on severity of coronary artery disease in acute MI. We found that ApoE33 genotypes were the most common genotype. This finding is universal for human populations [13]. In a recent study, in seven different localities of Turkey, ApoE3 was found to be the most common genotype. ApoE4 and ApoE2 allele incidences were 7,9% and 6,1%, respectively [14]. Besides, Singh et al. showed that the ApoE3 allele and ApoE33 genotype were most common in normal population and patients with angiographically diagnosed CAD [15, 16].

In the present study, there was no statistically significant difference among ApoE genotypes and severity of coronary artery disease by using the Gensini score. However, the patients with ApoE34 genotype and ApoE4 allele had high Gensini scores. As a result, these findings may be due to effects on plasma lipids. Both the levels of LDL-C and TC were observed to be higher ApoE34 genotype than the other genotypes.

ApoE4 allele is also associated with increased ApoB and cholesterol levels and decreased ApoE levels. In addition to coronary artery disease, ApoE gene polymorphisms were in relationship with many other diseases, including Alzheimer's disease, DM, Parkinson's disease, renal disease, and stroke [7, 17–20]. Similarly, the ApoE4 allele has been associated with higher levels of TC and LDL-C in several studies [21]. Also, Zende et al. found that there were significantly higher levels of serum TC, LDL-C, and triglyceride in patients with ApoE4 allele [22]. However, some researchers did not find any relationships between ApoE4 alleles and lipid levels [23]. There were some other studies where the ApoE4 allele was demonstrated to be a risk factor for atherosclerosis, and high levels of TC and LDL-C were suggested to determine the severity of CAD in patients with ApoE44 genotype [24]. In a meta-analysis, linear relations of ApoE genotypes with coronary risk were reported [25]. Çiftdoğan et al. evaluated the association of ApoE gene polymorphism and lipid levels in children with family history of premature CAD. The E4 allele may be associated with increased risk for development of atherosclerosis by elevated levels of TC [26]. Mendes-Lana et al. demonstrated that the presence of ApoE2 may be a protective effect for CAD, and the presence of ApoE4 may show an enhanced risk of dyslipidemia [27]. In addition, a meta-analysis published in 2014 showed that ApoE4 allele may be a risk factor for CAD [28]. Our study suggested the patients with ApoE4 allele had high plasma cholesterol and LDL-C levels.

The main limitations of this study are the limited number of the patients and the lack of a control group.

## 5. Conclusion

In this study, we found that there was no statistically significant difference between ApoE genotypes and severity of coronary artery disease by using the Gensini score in patients with acute MI in the southeast region of Turkey. But lipid profile was shown to be deteriorated in the patients with ApoE34 genotype and ApoE4 allele carriers. These findings suggest that E4 allele carriers may be genetically prone to atherosclerosis.

## Figures and Tables

**Table 1:** Demographic characteristics and distribution of lipid parameters of the patients according to ApoE genotype.

	ApoE22 *n*: 1	ApoE23 *n*: 17	ApoE33 *n*: 80	ApoE34 *n*: 40	*p*
Age	57	57,2 ± 9	57,5 ± 11	59 ± 55	0,74
Sex (F/M) (*n*)	0/1	3/14	16/64	12/28	0,88
HT (*n*)	0	2	24	1	0,89
DM (*n*)	0	2	11	1	0,89
Family history (*n*)	0	2	9	0	0,26
Smoking (*n*)	1	8	42	5	0,88
Gensini score	14	44,8 ± 28	35,6 ± 20	47,2 ± 26	0,13
Multivessel disease (*n*)					
1 vessel	1	6	41	16	
2 vessels	—	5	22	13	0,24
3 vessels	—	6	17	11	
Infarct-related artery *n*,					
LAD	1	8	31	16	0,29
RCA	—	3	11	12	
CX	—	6	38	12	
Glucose (mg/dL)	145	220 ± 113	159 ± 69	164 ± 60	0,17
Total cholesterol (mg/dL)	200	177 ± 51	183 ± 42	243 ± 91	0,10
Triglyceride (mg/dL)	171	180 ± 113	155 ± 99	152 ± 121	0,22
LDL (mg/dL)	124	111 ± 32	116 ± 31	156 ± 39	0,01
HDL (mg/dL)	33	36 ± 10	37,7 ± 7	42 ± 20	0,28

CX: circumflex artery, DM: diabetes mellitus, F: female, HDL: high density lipoprotein, HT: hypertension, LAD: left anterior descending, LDL: low density lipoprotein, M: male, and RCA: right coronary artery.

**Table 2:** Lipid and glucose levels in the patients with or without ApoE4 allele carrier.

	ApoE4 allele(+) *n*: 40	ApoE4 allele(−) *n*: 98	*p*
Glucose (mg/dL)	164 ± 60	169 ± 81	0,79
Total cholesterol (mg/dL)	243 ± 91	183 ± 44	0,03
Triglyceride (mg/dL)	152 ± 121	159 ± 101	0,51
LDL (mg/dL)	156,5 ± 39	115 ± 31	0,001
HDL (mg/dL)	42,2 ± 20	36,5 ± 8	0,27
Gensini score	47,2 ± 27	37 ± 22	0,22

## References

[1] Wang X. L., McCredie R. M., Wilcken D. E. L. (1995). Polymorphisms of the apolipoprotein E gene and severity of coronary artery disease defined by angiography. *Arteriosclerosis, Thrombosis, and Vascular Biology*.

[2] Stengerd J. H., Zerba K. E., Pekkanen J., Ehnholm C., Nissinen A., Sing C. F. (1995). Apolipoprotein E polymorphism predicts death from coronary heart disease in a longitudinal study of elderly Finnish men. *Circulation*.

[3] Wilson P. W. F., Schaefer E. J., Larson M. G., Ordovas J. M. (1996). Apolipoprotein E alleles and risk of coronary disease: a meta-analysis. *Arteriosclerosis, Thrombosis, and Vascular Biology*.

[4] Kolovou G. D., Anagnostopoulou K. K. (2007). Apolipoprotein E polymorphism, age and coronary heart disease. *Ageing Research Reviews*.

[5] Anoop S., Misra A., Meena K., Luthra K. (2010). Apolipoprotein E polymorphism in cerebrovascular & coronary heart diseases. *Indian Journal of Medical Research*.

[6] Kesäniemi Y. A., Ehnholm C., Miettinen T. A. (1987). Intestinal cholesterol absorption efficiency in man is related to apoprotein E phenotype. *Journal of Clinical Investigation*.

[7] Mahley R. W., Rall S. C. (2000). Apolipoprotein E: far more than a lipid transport protein. *Annual Review of Genomics and Human Genetics*.

[8] Davignon J., Gregg R. E., Sing C. F. (1988). Apolipoprotein E polymorphism and atherosclerosis. *Arteriosclerosis*.

[9] Siest G., Pillot T., Regis-Bailly A. (1995). Apolipoprotein E: an important gene and protein to follow in laboratory medicine. *Clinical Chemistry*.

[10] Knouff C., Hinsdale M. E., Mezdour H. (1999). Apo E structure determines VLDL clearance and atherosclerosis risk in mice. *The Journal of Clinical Investigation*.

[11] Ward H., Mitrou P. N., Bowman R. (2009). APOE genotype, lipids, and coronary heart disease risk: a prospective population study. *Archives of Internal Medicine*.

[12] Gensini G. G. (1983). A more meaningful scoring system for determining the severity of coronary heart disease. *The American Journal of Cardiology*.

[13] Burman D., Mente A., Hegele R. A., Islam S., Yusuf S., Anand S. S. (2009). Relationship of the ApoE polymorphism to plasma lipid traits among South Asians, Chinese, and Europeans living in Canada. *Atherosclerosis*.

[14] Mahley R. W., Palaoğlu K. E., Atak Z. (1995). Turkish heart study: lipids, lipoproteins, and apolipoproteins. *Journal of Lipid Research*.

[15] Singh P. P., Singh M., Bhatnagar D. P., Kaur T., Gaur S. P. (2008). Apolipoprotein E polymorphism and its relation to plasma lipids in coronary heart disease. *Indian Journal of Medical Sciences*.

[16] Singh P. P., Singh M., Mastana S. S. (2006). APOE distribution in world populations with new data from India and the UK. *Annals of Human Biology*.

[17] Wilson P. W. F., Myers R. H., Larson M. G., Ordovas J. M., Wolf P. A., Schaefer E. J. (1994). Apolipoprotein E alleles, dyslipidemia, and coronary heart disease. The Framingham offspring study. *The Journal of the American Medical Association*.

[18] Raber J., Huang Y., Ashford J. W. (2004). ApoE genotype accounts for the vast majority of AD risk and AD pathology. *Neurobiology of Aging*.

[19] Dergunov A. D. (2011). Apolipoprotein E genotype as a most significant predictor of lipid response at lipid-lowering therapy: mechanistic and clinical studies. *Biomedicine & Pharmacotherapy*.

[20] Khan T. A., Shah T., Prieto D. (2013). Apolipoprotein E genotype, cardiovascular biomarkers and risk of stroke: systematic review and meta-analysis of 14,015 stroke cases and pooled analysis of primary biomarker data from up to 60,883 individuals. *International Journal of Epidemiology*.

[21] Haddy N., De Bacquer D., Chemaly M. M. (2002). The importance of plasma apolipoprotein E concentration in addition to its common polymorphism on inter-individual variation in lipid levels: results from Apo Europe. *European Journal of Human Genetics*.

[22] Zende P. D., Bankar M. P., Kamble P. S., Momin A. A. (2013). Apolipoprotein E gene polymorphism and its effect on plasma lipids in arteriosclerosis. *Journal of Clinical and Diagnostic Research*.

[23] Attila G., Acartürk E., Eskandari G. (2001). Effects of apolipoprotein E genotypes and other risk factors on the development of coronary artery disease in Southern Turkey. *Clinica Chimica Acta*.

[24] Lehtinen S., Lehtimaki T., Sisto T., Salenius J. P. (1995). Apolipoprotein E polymorphism, serum lipids, myocardial infarction and severity of angiographically verified coronary artery disease in men and women. *Atherosclerosis*.

[25] Bennet A. M., Di Angelantonio E., Ye Z. (2007). Association of apolipoprotein e genotypes with lipid levels and coronary risk. *The Journal of the American Medical Association*.

[26] Çiftdoğan D. Y., Coskun S., Ulman C., Tıkız H. (2012). The association of apolipoprotein E polymorphism and lipid levels in children with a family history of premature coronary artery disease. *Journal of Clinical Lipidology*.

[27] Mendes-Lana A., Pena G. G., Freitas S. N. (2007). Apolipoprotein E polymorphism in Brazilian dyslipidemic individuals: Ouro Preto Study. *Brazilian Journal of Medical and Biological Research*.

[28] Zhang M.-D., Gu W., Qiao S.-B., Zhu E.-J., Zhao Q.-M., Lv S.-Z. (2014). Apolipoprotein e gene polymorphism and risk for coronary heart disease in the chinese population: a meta-analysis of 61 studies including 6634 cases and 6393 controls. *PLoS ONE*.

